# Insomnia and anxiety during the COVID-19 pandemic. a retrospective study on anxiety and sleep disorders among psychiatric patients admitted at „elisabeta doamna” hospital galati, romania

**DOI:** 10.1192/j.eurpsy.2021.772

**Published:** 2021-08-13

**Authors:** I.D. Rădulescu, C. Moraru, P. Nechita, A.B. Ciubară, A. Ciubară

**Affiliations:** 1 Corresponding Author, Psychiatrist, “Elisabeta Doamna” Psychiatric Hospital, Galati, Romania; 2 Resident Psychiatrist, Insitute of Psychiatry “Socola”, Iasi, Romania; 3 Corresponding Author, Md, Ph.d., Senior Psychiatrist, Insitute of Psychiatry “Socola”, Iasi, Romania; 4 Md, Ph.d, Associate Professor, Faculty of Medicine and Pharmacy, University “Dunarea de Jos”, Galati, Romania; 5 Md, Ph.d., Hab. Professor, Faculty of Medicine and Pharmacy, University “Dunarea de Jos” Head of Psychiatry Department, Senior Psychiatrist at ”Elisabeta Doamna” Hospital, Galati, Romania

**Keywords:** anxiety disorders, sleep disorders, pandemic, COVID-19

## Abstract

**Introduction:**

In public mental health there are widespread concerns about the effects of the Covid-19 pandemic on psychiatric patients. Anxiety and sleep disorders are the focal point in admissions for psychiatric care in individuals that are impacted by these changes.

**Objectives:**

The purpose of this study was to investigate the impact of the COVID-19 pandemic on the prevalence of anxiety and sleep disorders among the patients admitted to our hospital. The state of pandemia was declared on the 11^th^ of March but it had already become a main stream media subject in our country at the beginning of the month.

**Methods:**

A retrospective study was performed at the Psychiatric Hospital ‘Elisabeta Doamna’ Galati, using the exact same period, between 01.03 and 30.09, in both 2019 and 2020. ICD-10 criteria were used and pacients with either F41.x or F51.x as discharge diagnosis were included.

**Results:**

In total, 7638 cases were admitted during the period in 2019, of which 621 (8,13%) had anxiety disorders and 225 (2,94%) sleep disorders. In comparison in 2020 out of 4050 admitted patients, the number had risen to 1003 (24,76%) anxiety disorders and 388 (9,58%) sleep disorders.

**Conclusions:**

Analysis of the data shows a three times increase in the percentage of both classes of disorders among our patients. Even considering the lower admition rates, there is a clear shift in the general profile of our average pacient and this has to be taken into consideration in the long and short term treatment of any psychiatric patient.

